# An unusual case of secondary syphilis misdiagnosed as allergic dermatitis for 2 years

**DOI:** 10.1002/ccr3.3229

**Published:** 2020-08-13

**Authors:** Hong‐wei Wang, Ren‐he Chen, Ru‐zhi Zhang

**Affiliations:** ^1^ Department of Dermatology The Third Affiliated Hospital of Soochow University Changzhou China; ^2^ LiYang Institute of Dermatology LiYang China

**Keywords:** dermatitis, syphilis

## Abstract

Dermatologists should be aware that the clinical manifestations of syphilis are very complex and changeable. Unilaterally distributed skin lesions and painless lip ulcers may also be the clinical manifestations of secondary syphilis.

## Graphical abstract

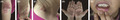

## 1 INTRODUCTION

We report a patient who had been misdiagnosed as allergic reactions for 2 years until new lesions appeared in the neck, palms, and soles of the patient. Combined with the newly discovered history of sexual contact, serological, and pathological examination, the final diagnosis of secondary syphilis was confirmed.

Syphilis is a chronic infection caused by *Treponema pallidum*, first recognized in the 15th century in Europe, with an increasing worldwide incidence.^1^ It is mainly sexually transmitted, although it can develop through hematological or vertical pathways.^2^ Depending on the number and virulence of the *Treponema* and the host response, the incubation period is usually 3‐4 weeks after contact, although it can vary from 10 to 90 days.^3^ Syphilis has multiple clinically active phases with intervening latency periods. This disease earned the name of “The great imitator” for its varied clinical manifestations.^4^

## 2 CASE REPORT

A 48‐year‐old married woman had some recurrent rashes in her right axillary area and cubital fossa for 2 years. Recently, several isolated red nodules developed on her right neck, with erythema on both palms and soles, with mild itching. During the past 2 years, she had been diagnosed several times with allergic dermatitis and was treated with oral antihistamines and mild potent topical corticosteroids, along with instructions to avoid scratching. Within this period, the patient used antibiotics irregularly due to upper respiratory infections. Her lesions had temporarily subsided but soon recurred at the original sites. Furthermore, several red asymptomatic papules and nodules developed on the right nape of her neck along with some erythema with mild desquamation on both palms and soles.

In the previous month, the patient's skin lesions had been increasing significantly. External application of a potent corticosteroid cream was ineffective. Therefore, the patient came to our hospital for a dermatology consultation. She stated that she was in good health, denied a history of fever and nonmarital contact, and had no history of blood transfusions.

**FIGURE 1:**
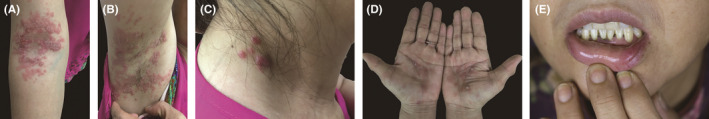
Clinical manifestation before treatment. Well‐demarcated infiltrating erythematous papules and plaques, with or without some desquamation on the surface, distributed in her right axillary area (A) and cubital fossa (B). Four inflamed bright red papules and nodules on the right nape of her neck (C). Several “copper” pigmented macules were seen on both palms (D). An infiltrated dark erythema on her lower lip (E)

Dermatological examination found well‐demarcated infiltrating erythematous papules and plaques, with or without some desquamation on their surface, at her right axillary area (Figure 1A) and cubital fossa (Figure 1B). There were four inflamed bright red papules and nodules on the right nape of her neck (Figure 1C). Several “copper” pigmented macules were seen on both palms and soles (Figure 1D). Mucosal examination revealed an infiltrated dark erythema on her lower lip (Figure 1E). The patient reported that she had a painless ulcer on her lower lip, which had healed spontaneously. There were no lesions in her oral cavity, perianal region, groin folds, labia majora, and pubic region. Physical examination showed swelling of the superficial lymph nodes in her right axillary area. No skin lesions or lymphadenopathy was found in other areas.

**FIGURE 2:**
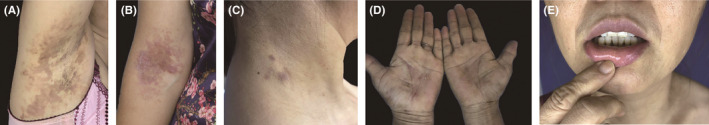
Clinical manifestation after treatment. The dark red patches and plaques distributed in the right axillary area (A) and cubital fossa (B) had regressed. Four dark red papules and nodules on the right nape of her neck (C). Several dark red pigmented macules were seen on both palms (D). No obvious damage remained on her lip (E)

Tests for human immunodeficiency virus, hepatitis B, hepatitis C, and patch tests were all negative. Serologic testing for rapid plasma reagin (RPR) was positive at a titer of 1:32, and a test for *T pallidum* particle agglutination (TPPA) was also positive. Repeated questioning about the possible source of infection revealed that her husband was diagnosed with syphilis 2 days ago. She and her husband had frequent unprotected practice, including oral sex and kissing on the mouth. Her husband had a positive TPPA test, had RPR at a titer of 1:16, but was being treated. He refused to come to the hospital for further inquiry. A skin biopsy was performed from the lesion on the nape of her neck and revealed acanthosis and hyperkeratosis in the epidermis, with a perivascular mixed inflammatory cell infiltrate composed mainly of plasma cells and lymphocytes in the dermis. The diagnosis of secondary syphilis was established by the clinical, laboratory, and microscopic findings. The patient received two consecutive weekly injections of intramuscular benzathine penicillin G 2.4 million units after sensitivity testing, after which the skin lesions slowly regressed (Figure 2).

## 3 DISCUSSION

Secondary syphilis is the result of the hematogenous dissemination of treponemes.^5^ This stage is characterized by an extensive maculopapular rash on the skin and multiple lesions in mucosal surfaces and internal organs. Therefore, the patients may have symptoms such as fever, myalgia, headache, arthralgia, and generalized lymphadenopathy. The appearance of lesions and systemic symptoms depends on the virulence of the treponema and the response of the host. The most common cutaneous presentation is generalized, nonpruritic, symmetrical macular eruptions, which are frequently distributed on the trunk, palms, and soles, called syphilitic rosette.^6^ Individual lesions, especially on the palms and soles, are usually “copper” pigmented. However, previous studies have demonstrated that up to 29.6% of cutaneous lesions of secondary syphilis may display atypical morphologies, which include nodular, nodulo‐ulcerative, lichenoid popular, annular, pustular, framboesiform, corymbose, leukoderma, and chancriform presentations.^7^ In the present case, the lesions displayed an itchy erythema and papules with a small amount of desquamation and were confined to the right axillary area and cubital fossa. She had been misdiagnosed as an allergic reaction for the past 2 years. Recently, new lesions had developed on her neck, palms, and soles, and she was definitively diagnosed as secondary syphilis based on serological and pathological examination.

Although various clinical manifestations of syphilis have been described for many centuries, our case has the following unique features: (a) Skin lesions were unilaterally localized and distributed, except for the typical skin lesions on both palms and soles; all other lesions were located on one side of the body, which cannot be explained by coincidence. In the literature, no similar reports have been published. (b) Skin lesions appeared as scaly erythemas, papules, and bright red nodules, and the pigmentation remaining after treatment had subsided. The long duration, hypersensitive reaction, irregular use of oral antibiotics, and topical corticosteroids, may be partially to blame. (c) The neglected painless ulcer on her lower lip. Mucosal plaques, which are usually slightly elevated and covered by membranes, are the most common manifestation of secondary syphilis, preferentially located on the lips, tongue, buccal mucosa, and palate.^8^ However, ulcerated lesions with irregular and whitish borders may also be observed. Some practices may transmit *T pallidum*, for example, through oral sex, kissing on the mouth and sharing toothbrushes. This patient and her husband with syphilis had frequently unprotected practices, including oral sex and kissing, which may explain the source of her infection.

The diagnosis of secondary syphilis is very important, since if undiagnosed and consequently untreated, it may eventually lead to tertiary syphilis,^9^ which is a life‐threatening condition. Dermatologists are challenged by the unusual presentations from secondary syphilis and need to maintain a high index of clinical suspicion as the primary stage may go undiagnosed.

## CONFLICT OF INTEREST

None declared.

## AUTHOR CONTRIBUTION

H‐wW: is principal author, R‐hC: is supporting author, R‐zZ: is principal investigator.
